# Delayed visual loss due to radiation retinopathy

**DOI:** 10.12669/pjms.322.9221

**Published:** 2016

**Authors:** Salih Uzun, Sami Toyran, Fahrettin Akay, Fatih C. Gundogan

**Affiliations:** 1 Dr. Salih Uzun, Department of Ophthalmology, Etimesgut Military Hospital, Ankara, Turkey; 2 Dr. Sami Toyran, Department of Ophthalmology, Izmir Military Hospital, Izmir, Turkey; 3 Dr. Fahrettin Akay, Department of Ophthalmology, Izmir Military Hospital, Izmir, Turkey; 4 Fatih Cakir Gundogan, Associate Professor, Department of Ophthalmology, GATA Medical School, Ankara, Turkey

**Keywords:** Nasopharyngeal carcinoma, Radiation maculopathy, Radiation retinopathy, Radiotherapy

## Abstract

Radiation retinopathy remains a devastating cause of visual morbidity in patients undergoing radiation for globe, orbit, and head and neck malignancies. A 65-year-old female was admitted with the complaint of low vision in the right eye for two months. Best corrected visual acuity was 20/32 in the right eye and 20/25 in the left eye. Slit lamp examination was normal in both eyes. Fundoscopic examination revealed perifoveolar hard exudates, paramacular microhemorrhages, telangiectasias, and macular degeneration in both eyes. Fundus florescein angiography showed enlargement of the foveal avascular zone, perifoveal capillary telangiectasia, and widespread venous beading bilaterally. Optical coherence tomography revealed bilateral cystoid macular edema. The prediagnosis of diabetic retinopathy was not confirmed because of the absence of diabetes mellitus after endocrinologic evaluation. Detailed medical history explored external beam radiotherapy to the head and neck region for nasopharyngeal cancer 10 years ago. The ultimate diagnosis was radiation retinopathy.

## INTRODUCTION

Radiation retinopathy is a delayed, well-known complication of radiotherapy. It appears due to endothelial cell damage resulting in chronic progressive vasculopathy of the retinal capillaries.^1^ The manifestations are similar to diabetic retinopathy and include endothelial sloughing, neovascularization, optic neuropathy, dilation of capillaries, increased vascular permeability, thrombosis, retinal cotton wool spots and exudates, hemorrhages, and macular edema.^1^-^3^

In this case report, we aimed to present a case with radiation retinopathy that developed 10 years after external beam radiotherapy due to nasopharyngeal carcinoma.

## CASE REPORT

**Fig.1:**
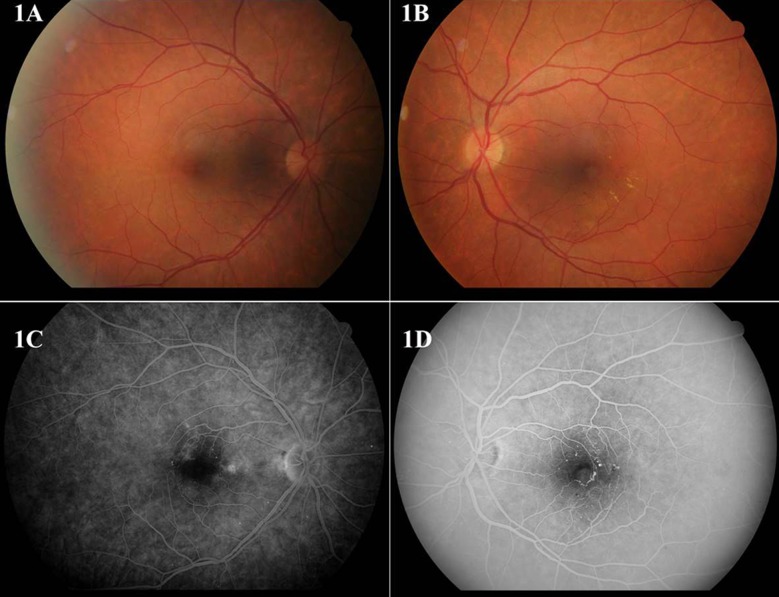
Color fundus photography (1A, 1B) and fundus fluorescein angiography (1C, 1D) of both eyes.

A 65-year-old female was admitted with the complaint of low vision in her left eye with two months history. The patient did not have any history of a systemic or ophthalmic disease and ocular trauma. Best corrected visual acuity was 20/32 in the right eye and 20/25 in the left eye. The slit lamp examination was irrelevant in both eyes. Intraocular pressure was 13 mmHg bilaterally. Fundoscopic examination showed perifoveolar hard exudates and telangiectasias in the left eye (Fig.1A, 1B). Fundus fluorescein angiography (FFA) revealed bilateral perifoveal capillary telangiectasias and perifoveolar capillary beading in the left eye (Fig.1C, 1D). The patient was prediagnosed with diabetic retinopathy and consulted to endocrinology clinic for the diagnosis of diabetes mellitus.

Endocrinologic evaluation showed serum fasting blood glucose level of 85 mg/dl, 120-minute postprandial glucose level of 96 mg/dl and HbA1c level of 6.1%. The patient was not diagnosed with diabetes. A detailed questioning of medical history revealed external head and neck radiotherapy for nasopharyngeal carcinoma 10 years ago. In the light of clinical findings and radiotherapy history, the patient was diagnosed as radiation retinopathy.

## DISCUSSION

Malignant and sometimes benign neoplasms of the head and neck, neoplastic (optic nerve sheath meningioma) and non-neoplastic (Graves’ ophthalmopathy) orbital disorders may be treated with radiotherapy.^1^, ^4^ Radiotherapy may also be used to treat non-neoplastic intraocular lesions such as wet type age-related macular degeneration.^1^ Radiotherapy for an extraocular malignant disease may result in significant radiation damage to the eye.^1^, ^5^

Radiation retinopathy is directly correlated with total dose of radiation, fraction size, presence of concurrent chemotherapy, and comorbid conditions such as hypertension, diabetes, and collagen vascular diseases.^2^ The threshold for radiation retinopathy is 45-50 Gy (1.8–2 Gy daily fractions). More than 50% of the patients that receive 65 Gy to healthy retina will develop radiation retinopathy.^1^, ^4^ It is supposed that a radiation dose less than 35 Gy rarely causes retinopathy.^1^ The most radiosensitive region of the retina is the posterior polar region.^1^ Radiation retinopathy usually begins within 6-12 months after completion of radiotherapy, and once it appears, it is irreversible and there are no known effective treatment modality.^6^

Detection of vascular occlusive changes, vascular dilatation, emergence of collaterals, and microaneurysmal changes in the capillaries are the microscopic determinants of the disease.^1^, ^2^ Radiation retinopathy is characterized by parenchymal inflammation, leukocyte pooling in the capillaries, glial hypertrophy, necrotic and gliotic changes, neuronal swelling and degeneration.^6^ Microscopic characteristics of the final phase of radiation retinopathy are co-existence of regenerated vessels and ischemic fields.^7^ FFA shows capillary nonperfusion, and this finding is the gold standard to demonstrate radiation retinopathy.^7^ Our patient had enlargement of the foveal avascular zone, perifoveal capillary telangiectasia and widespread venous beading in both eyes on FFA.

Radiation optic neuropathy is also seen frequently. Radiation scleral necrosis is less frequent possibly due to resistance of scleral collagen to radiation.^1^ Those complications of radiotherapy may cause blindness as a result of radiation retinopathy, optic neuropathy, or enucleation of the eye due to scleral necrosis.^1^

The only preventive measure is to keep the retinal radiation dose below the tolerance limits, however this may not be realistic.^6^ Radiation retinopathy has been treated with multiple off-label treatments, including intravitreal corticosteroids, intravitreal anti-VEGF therapy, and laser therapy with variable success rates.^4^, ^8^, ^9^ Variable efficacy of treatment modalities may be secondary to different foveal exposures of the globe in different treatment regimens.^1^, ^4^ Patients with macular edema also have a vast range of ocular comorbidities such as optic neuropathy, macular ischemia, neovascularization, and cataract; therefore comparison among the studies is difficult.^3^

Clinical differential diagnosis of radiation retinopathy include diabetic retinopathy, previous multiple obstructions of retinal veins, and retinal telangiectasia due to various reasons. The findings of our patient were in concordance with diabetic retinopathy, and we supposed that our patient had an undiagnosed diabetes mellitus. The most important entity in differential diagnosis is the presence of previous radiotherapy in the history of the patient. Therefore medical history should be carefully questioned.
